# Role of Domain–Domain Interactions on the Self-Association and Physical Stability of Monoclonal Antibodies: Effect of pH and Salt

**DOI:** 10.1021/acs.jpcb.3c03928

**Published:** 2023-09-26

**Authors:** Amy Y. Xu, Marco A. Blanco, Maria Monica Castellanos, Curtis W. Meuse, Kevin Mattison, Ioannis Karageorgos, Harold W. Hatch, Vincent K. Shen, Joseph E. Curtis

**Affiliations:** † Department of Chemistry, Louisiana State University, Baton Rouge, Louisiana 70803, United States; ‡ Discovery Pharmaceutical Sciences, Merck Research Laboratories, Merck & Co., Inc, West Point, Pennsylvania 19486, United States; § Institute for Bioscience and Biotechnology Research, University of Maryland, Rockville, Maryland 20850, United States; ∥ NIST Center for Neutron Research, National Institute of Standards and Technology, Gaithersburg, Maryland 20899, United States; ⊥ Biomolecular Measurement Division, National Institute of Standards and Technology, Gaithersburg, Maryland 20899, United States; # Malvern Panalytical, Westborough, Massachusetts 01581, United States; 7 Chemical Sciences Division, Material Measurement Laboratory, National Institute of Standards and Technology, Gaithersburg, Maryland 20899, United States

## Abstract

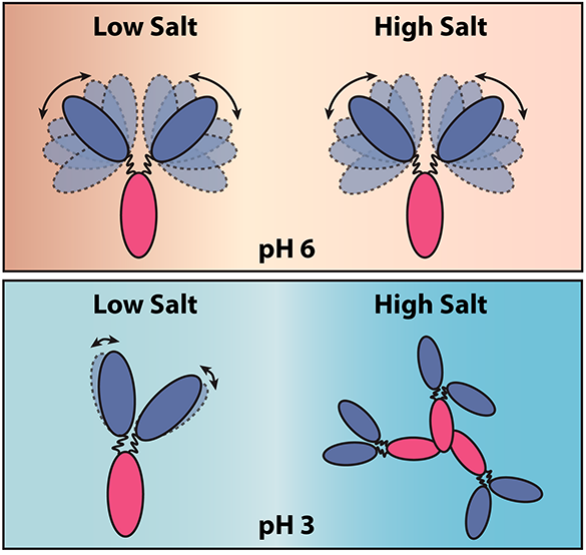

Monoclonal antibodies (mAbs) make up a major class of biotherapeutics with a wide range of clinical applications. Their physical stability can be affected by various environmental factors. For instance, an acidic pH can be encountered during different stages of the mAb manufacturing process, including purification and storage. Therefore, understanding the behavior of flexible mAb molecules in acidic solution environments will benefit the development of stable mAb products. This study used small-angle X-ray scattering (SAXS) and complementary biophysical characterization techniques to investigate the conformational flexibility and protein–protein interactions (PPI) of a model mAb molecule under near-neutral and acidic conditions. The study also characterized the interactions between Fab and Fc fragments under the same buffer conditions to identify domain–domain interactions. The results suggest that solution pH significantly influences mAb flexibility and thus could help mAbs remain physically stable by maximizing local electrostatic repulsions when mAbs become crowded in solution. Under acidic buffer conditions, both Fab and Fc contribute to the repulsive PPI observed among the full mAb at a low ionic strength. However, as ionic strength increases, hydrophobic interactions lead to the self-association of Fc fragments and, subsequently, could affect the aggregation state of the mAb.

## Introduction

Monoclonal antibodies (mAbs) have emerged as the primary class of biotherapeutics in the pharmaceutical industry owing to their high efficacy and specificity in treating various medical conditions.^1,2^ Despite the widespread use of mAbs, the development of these molecules continues to pose a challenge due to the limited understanding of the physics that influences product stability. Ensuring that mAbs remain in their functional native state without any physical or chemical alterations during manufacturing, storage, and delivery is crucial for their effectiveness and safety, as well as for meeting commercial and regulatory requirements.^3^

Developing effective mAb therapeutics requires careful consideration of the stabilizing solution conditions and formulations. Different environmental factors such as pH, temperature, and the type and concentration of excipients can significantly impact the nature, strength, and range of intra- and intermolecular interactions.^4^ At high-concentrations (e.g., >100 mg/mL), the stabilizing conditions can be compromised, as the average interprotein distances are on the order of the molecular dimensions, enhancing protein–protein interactions (PPI) and triggering other physical effects such as crowding and multibody interactions.^5^ Consequently, instability issues such as elevated viscosity, opalescence, and phase separation may emerge alongside the aforementioned problems, leading to further challenges in mAb development. Thus, understanding the relationship among solution conditions, protein interactions, and stability is crucial to identify suitable mAb formulations.

The physical stability of proteins is dependent on PPI, which are weak and result from a combination of forces such as electrostatic, hydrophobic, van der Waals, steric, hydrogen-bonding, and dipole–dipole interactions.^6^ Protein surface anisotropy refers to the uneven distribution of chemical groups on the protein surface resulting in regions with varying charge and hydrophobicity.^7^ Protein anisotropy plays a pivotal role in determining the nature of PPI among protein molecules and hence affects the physical stability of proteins in different solution environments.^2,8,9^ On the molecular level, mAbs are multidomain proteins connected by a flexible hinge region. The flexibility of the hinge region influences the relative orientations of the protein domains and, thus, the accessibility of local surface regions on mAb molecules, making them more or less accessible for various intermolecular interactions. In addition to conformational flexibility, understanding domain–domain interactions is also necessary for gaining insights into PPI among mAb molecules and developing models that can accurately predict their behavior. By characterizing the association propensity of individual domains, one can identify potential aggregation-prone sequences that contribute to PPI and design stable mAbs.^10^ Furthermore, knowledge of domain–domain interactions can guide the development of coarse-grained models to predict PPI among mAbs, where Fab and Fc domains are treated as either individual beads or a collections of beads with various interaction propensity.^5,11−16^ Despite significant progress, more experimental data on domain–domain interactions are needed to provide experimental results to refine the force fields and surface characteristics of beads for improved simulation results.

During the development of mAb products, mAb molecules are frequently exposed to buffer conditions with a low pH and high salt concentration. For example, salt is used during the protein purification process to prevent proteins from adhering to the HPLC column resin.^17^ The purification process using protein A columns exposes mAbs to an acidic pH environment. Moreover, therapeutic mAbs are often formulated at slightly acidic pH conditions deviating from their isoelectric points.^18^ Therefore, understanding how mAb molecules behave under low pH and high salt conditions is key not only for acquiring fundamental insights into the impacts of pH and ionic strength on the conformation and PPI of mAbs, but also for facilitating the rational design of mAbs that can remain stable during the manufacturing process. In this study, we utilized NIST reference antibody RM8671 (NISTmAb) as a model antibody to investigate the structure, conformational flexibility, and PPI among mAb molecules in solutions with low pH and high ionic strength. In addition to measuring the PPI among NISTmAb molecules, we prepared Fab and Fc fragments by papain digestion of the mAb molecule and characterized the charge and PPI among the individual fragments to determine the domain–domain interactions under similar buffer conditions. Our findings indicate that the conformational flexibility of the NISTmAb changes significantly with solution pH and that the domain–domain interactions play a significant role in determining the overall PPI among the full antibody molecules.

## Materials and Methods

Certain commercial equipment, instruments, or materials are identified in this paper to foster understanding. Such identification does not imply recommendation or endorsement by the National Institute of Standards and Technology, nor does it imply that the materials or equipment are necessarily the best available for the purpose.

### Sample Preparation

NIST reference antibody RM8671 (NISTmAb) and its cleaved Fab and Fc fragments were prepared in two different buffer solutions: (i) citrate/phosphate, pH 3–7 and (ii) glycine/HCl, pH 3. Unless otherwise indicated, all reactants had a level of purity not inferior to that of ACS grade. Stock phosphate/citrate buffer solutions were prepared by dissolving anhydrous citric acid and sodium phosphate dibasic (Fisher Scientific, Hampton NH) in Milli-Q water; added amounts of these reactants were calculated to achieve the target pH and phosphate/citrate concentration. All buffer solutions were prepared to a concentration no higher than 10 or 25 mM (for phosphate/citrate and glycine/HCl, respectively), while solutions were supplemented with NaCl (Fisher Scientific) to adjust ionic strength to the desired concentration. The unit M stands for the molar concentration of mol/L, whereas mM stands for the concentration of 10^–3^ mol/L.

### Antibody Cleavage

The Fab and Fc fragments of NISTmAb were cleaved by papain digestion. Briefly, per 1 mg/mL of NISTmAb sample was digested using a 0.01 mg/mL colloidal papain from papaya latex (Sigma-Aldrich, P3125) in a 100 mM sodium acetate buffer containing 1 mM EDTA and 40 mM cysteine at pH 5.5. The mAb and papain mixture was incubated at 37 °C for 2 h for optimal cleavage. The digested sample was then filtered using a 0.22 μm syringe filter to remove particulates and concentrated using a 10 kDa spin filter for further HPLC purification. To isolate the fragments from the nondigested full mAb, the concentrated digest was separated by size exclusion chromatography (SEC) using a Superdex 75, 10/300 GL column. The SEC buffer consisted of 100 mM sodium acetate with 150 mM NaCl at pH 5.5. The pH of the Fab and Fc fractions was adjusted to pH 8 by adding 1 M Tris (pH 10) buffer. The Fab and Fc were further separated by affinity chromatography via a protein A column. The purity of all fractions was checked by sodium dodecyl sulfate-polyacrylamide gel electrophoresis (SDS-PAGE). The concentrations for Fab and Fc fragments were calculated by their absorbance at 280 nm using extinction coefficients of 1.47 and 1.43 mL mg^–1^ cm^–1^ respectively.^19^ The molecular weights of Fab and Fc were 47.6 and 50.2 kDa, respectively.^19^

### Light Scattering

SLS and DLS measurements were performed on a Wyatt DynaPro Nanostar (Wyatt Technology Inc., CA) equipped with a solid-state laser (λ = 658 nm) and a 512-channel, multi-τ correlator with a sampling time of 100 ns. Scattering measurements were performed on 1.25 mL quartz cuvettes at 25 ± 0.3 °C. The scattered light intensity and its autocorrelation function were obtained at 90° for NISTmAb and its fragments at protein concentrations ranging from 0.5 to 10 mg/mL. Prior to light scattering measurements, samples were centrifuged at 4000 rpm for 15 min. SLS and DLS data were obtained by time-averaging the instantaneous scattering intensities and autocorrelation functions over a time window of 1 min for a given sample. At least four independent replicates of each protein concentration were measured to reduce statistical uncertainties in the results. Absolute values of scattered intensity (Rayleigh ratio, *R*_90_) were obtained by normalization with respect to toluene,^20^ and analyzed to obtain information about the osmotic second virial coefficient *B*_22_ and apparent molecular weight *M*_app_ via eq 1:^21,22^
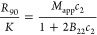
1where *K* = 4π^2^*n*^2^(d*n*/d*c*_*2*_*)*^2^/(*N*_A_λ^4^) is an optical constant. *N*_A_ is Avogadro’s number, *n* = 1.333 is the refractive index of the solution, and d*n*/d*c*_2_ = 0.185 is the derivative of *n* with respect to the protein concentration *c*_2_.

Fitting of SLS data vs protein concentration to eq 1 was performed for samples at equivalent solution conditions and over a concentration range that ensures a dilute regime (see below). That is, *B*_22_ is formally related to protein–protein interactions via eq 2:
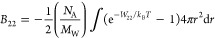
2with *k*_B_ and *T* being the Boltzmann constant and the absolute temperature, respectively. *M*_W_ is the true molecular weight of the protein. The factor *N*_A_/*M*_W_ is there to provide *B*_22_ with the same units as those in eq 1 (i.e., units of volume per mass). *W*_22_ is the grand-canonical potential of mean force, which corresponds to the strength of the interactions between two proteins averaged over the orientational degrees of freedom of both molecules and the spatial degrees of freedom of the solvent and any cosolute species in solution. Following eq 2, positive (negative) values of *B*_22_ are associated with net repulsive (attractive) PPI.

Because of the nature of *W*_22_ and eq 2, *B*_22_ is defined only in the limit of infinite dilution of protein (i.e., *c*_*2*_ → 0). To ensure that the employed data set preserves the “infinite dilution” condition, the concentration range used for fitting to eq 1 at every solution condition was selected to ensure the zero-*q* structure factor does not deviate more than 0.1 from unity (i.e., |S(*q* → 0) – 1| ≤ 0.1), as suggested previously.^22^ The zero-*q* structure factor is calculated from the SLS data as S(*q*→ 0) = *R*_90_*/(KM*_app_*c*_2_).^22^ For DLS experiments, the measured intensity autocorrelation function *g*^(2)^(*t*) was analyzed via the method of cumulants as
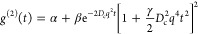
3where α is the average baseline (i.e., α = g^(2)^(∞)), β is the amplitude of the autocorrelation function (i.e., β = *g*^(2)^(0)), and *q* is the magnitude of the scattering vector, with *q* = 4πsin(θ/2)/λ and θ = 90°. *D*_c_ is the collective or mutual diffusion coefficient and in the infinite dilution limit is related to the hydrodynamic radius of the protein (*R*_h_) via the Stokes–Einstein equation, as *D*_c_ = *k*_B_*T*/(6πη_0_*R*_h_) with η_0_ being the viscosity of the solvent.

In eq 3, *D*_c_ corresponds to the first moment of the underlying distribution of diffusive decay times, while γ represents the polydispersity index of the solution and is such that γ*D*_c_^2^ is the second moment around the average for the same underlying distribution. In general, the polydispersity index is a dimensionless parameter associated with the width of the size distribution of protein species, where a value of γ ≤ 0.05 indicates effective monodisperse solutions. Note that the distribution of diffusive decay times can be mapped into the size distribution of proteins for negligible interactions between proteins and/or effectively dilute conditions.

In DLS experiments, the most relevant quantity to be calculated is the collective diffusion coefficient *D*_c_, as it contains information regarding the molecular size and the strength of the intermolecular interactions. Thus, DLS measurement of intermolecular interactions often relies on a series expansion in terms of protein concentration of *D*_c_, in which the first-order term of this expansion is related to PPI. That is
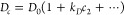
4where *D*_0_ = *k*_B_*T*/(3πη_0_σ) is the diffusion coefficient at infinite dilution, σ is the protein diameter, and *k*_B_, *T*, and η are defined above. *k*_D_ is the so-called DLS interaction parameter, and it is measured as the initial slope (i.e., as *c*_2_ is approaching zero) in a curve of *D*_c_ vs *c*_2_. Similar to *B*_22_, positive (negative) values of *k*_D_ are qualitatively related to repulsive (attractive) PPI. However, unlike *B*_22_, the value of *k*_D_ depends not only on “direct” protein interactions (i.e., the potential of mean force) but also on “indirect” hydrodynamic interactions (e.g., the effect of the Brownian motion on the behavior of proteins). Formally, *k*_D_ and *B*_22_ are related via:
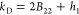
5with *h*_1_ being the offset between *B*_22_ and *k*_D_, which accounts for the effect of hydrodynamic forces on the strength of PPI. From a physics standpoint, *h*_1_ corresponds to the first-derivative of the zero-*q* hydrodynamic factor *H*(*q* → 0) with respect to protein concentration (i.e., *h*_1_ = d*H*(*q* → 0)/d*c*_2_

### Electrophoretic Light Scattering (ELS)

Protein mobility was measured by ELS using a Zetasizer Nano ZSP system (Malvern Panalytical, Westborough MA) at protein concentrations of 25 mg/mL for NISTmAb and its cleavage fragments. Prior to ELS measurements, samples were centrifuged at 3000 rpm for 15 min and double filtered through 0.02 μm Anotop filters (Fisher Scientific). ELS measurements were collected at 25 °C in a disposable capillary cell, using the diffusion barrier technique, with 30 μL sample injections. All measurements were collected in triplicate using the protein mobility measurement mode within the Zetasizer software, with the applied voltage and number of subruns set to auto-optimize. The electrophoretic mobility (μ_E_) was converted to effective charge (*Z*_eff_) via eq 6:
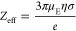
6where *e* is electronic charge, η is the sample viscosity, and σ is the protein diameter.

### Small-Angle X-ray Scattering (SAXS)

Small-angle X-ray scattering (SAXS) measurements were performed on a SAXSLab Ganesha instrument (SAXSLab, MA) at the Institute for Bioscience and Biotechnology Research, University of Maryland. Scattered photons were detected by using a two-dimensional Pilatus 300 K detector. Data reduction was performed using the RAW software.^23^ Approximately 100 μL of each sample was loaded into a 96-well plate. The plate was tape-sealed to prevent solvent evaporation. Using an automated liquid handling system, approximately 20 μL of sample was loaded into a 1.3 mm capillary. The capillary was thoroughly washed with water and 5% Hellmanex solution, and dried between each sample. Sample to detector distance was varied from 0.4 to 1.7 m, covering a *q*-range from 0.008 to 0.8 Å^–1^. The scattering vector *q* is defined as
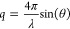
7Scattering measurements were performed for NISTmAb samples prepared with varying pH values and ionic strengths. In particular, NISTmAb samples prepared at 2 mg/mL were measured for each buffer condition. Higher concentration samples were also measured and used for structure factor analysis. The total scattering intensity *I*(*q*) of an ideal system consists of monodisperse, homogeneous, and isotropic dispersions of spherical particles can be expressed as
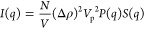
8where (*N*/*V*) and *V*_p_ are the number density and volume of scattering particles, respectively. Δρ is the difference in the scattering length density between the scattering particles and buffer background. *P*(*q*) is the form factor that is attributed to the shape and size of scattering objects, whereas *S*(*q*) is the structure factor resulting from interparticle interactions. Since mAb molecules are nonspherical and anisotropic, the *S*(*q*) measured from scattering experiments is referred to as the effective structure factor *S*(*q*)_eff_.^24−26^

In dilute samples, the distances between mAb molecules were sufficiently large so that the PPI values were negligible. The *P*(*q*) profiles of NISTmAb samples prepared in different buffer conditions were subjected to two types of analysis. One of them was a *P*(*r*) distribution analysis, where the distribution of the interatomic distances was obtained from the indirect Fourier transform of the scattering data. The *P*(*r*) distribution calculation was carried out using the GNOM program from the ATSAS software package.^27^ While the *P*(*r*) distribution analysis was useful for determining the size and probable shape of the protein molecules, the conformational flexibility of mAb molecules was further assessed using molecular simulation using SASSIE-web.^28,29^ Briefly, 68 956 nonoverlapping configurations of NISTmAb were generated by sampling the backbone dihedral angles of three amino acids on each heavy chain in the upper hinge region.^28,30^ The starting model of the intact NISTmAb molecule was built using the previously established method.^30^ The corresponding *P*(*q*) profile of each structure was calculated using the SasCalc module of SASSIE-web.^28,31^ Theoretical and experimental *P*(*q*) profiles were compared by assessing χ^2^ values calculated using the Chi-Square Filter module from SASSIE-web.^28^ Structures with χ^2^ values less than 5 were considered as good fits, i.e., the conformations that are most likely to be adopted by NISTmAb for a given pH and ionic strength. Subensembles with χ^2^ values less than 5 were represented as a density plot to demonstrate the space sampled. With increasing mAb concentration, the distances between individual mAb molecules were reduced to the point where PPI were present.

In this study, scattering profiles of 2 mg/mL mAb samples were used for the *P*(*q*) analysis. The effective structure factor *S*(*q*)_eff_ was extracted from the total scattering intensity measured from concentrated samples by removing the contribution from *P*(*q*) using the following equation:
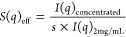
9where *s* is the scaling factor for the given concentration at which concentrated mAb samples were measured and is used to normalize the scattering profiles measured from various concentrations.^32,33^

Previous study from our group demonstrated that flexible mAb molecules can be treated as spheres at a larger length scale where configurational variations of mAbs do not perturb interparticle correlations.^25^ Therefore, the *S*(*q*)_eff_ measured from different buffer conditions were fitted using appropriate models to account for various repulsive and attractive interactions present among individual protein molecules.^25,26^ Three models were used to fit *S*(*q*)_eff_ profiles obtained from different samples, these include: (1) the hard sphere model, where the steric repulsion is considered to be the only intermolecular interaction; (2) the Hayter–Penfold model, where additional Coulomb repulsions between molecules are also considered; and (3) the Two–Yukawa model, where both attractive and repulsive interactions are taken into account.^25,34−37^

### Infrared Spectroscopy

The attenuated total reflection infrared spectra (ATR-FTIR) were obtained by using a Bruker Vertex 80 spectrometer (Billerica, MA). A DTGS detector was used to acquire data at 4 cm^–1^ resolution. The scans were 2 min long for both sample and background and had a 5 kHz sampling rate. The protein solutions were sampled at a 45° angle of incidence by using a Pike Technologies VeeMax II ATR accessory with a 45° ZnSe crystal (Madison, WI). For each of the tested protein solutions, 6 sample and 6 buffer scans were acquired relative to the empty crystal and averaged to produce the spectra. Buffer subtraction, data manipulation, and export were carried out with Bruker Opus 7.5 software. Spectral derivatives were determined using Opus 7.5 after the spectra were min-max normalized between 1720 and 1600 cm^–1^.

### Circular Dichroism

Far- and near-UV circular dichroism spectra (far- and near-CD, respectively) were measured by using an Applied Photophysics Chirascan V100 spectrometer (Leatherhead, Surrey, UK). Far-UV CD spectra were collected for samples at 1 mg/mL in the wavelength range of 190–250 nm with a bandwidth of 0.5 nm, using a demountable quartz cell with 0.1 mm path length. Similarly, samples at 1 mg/mL were used for obtaining near-CD spectra in the range of 240–360 nm with a bandwidth of 1 nm and using a micro quartz cell with 10 mm path length. For each sample, reported CD spectra were calculated from the average of at least five separate scans, which were taken using an acquisition time of 4 s and 10 s for far- and near- CD data, respectively. Additionally, spectra for the different buffers were measured to perform baseline subtraction. Data were processed using Chirascan Pro-Data and Pro-Data Viewer, version 4.4.0.

## Results

### Effects of pH and Ionic Strength on the Size and Charge of NISTmAb and Its Fragments

**Figure 1:**
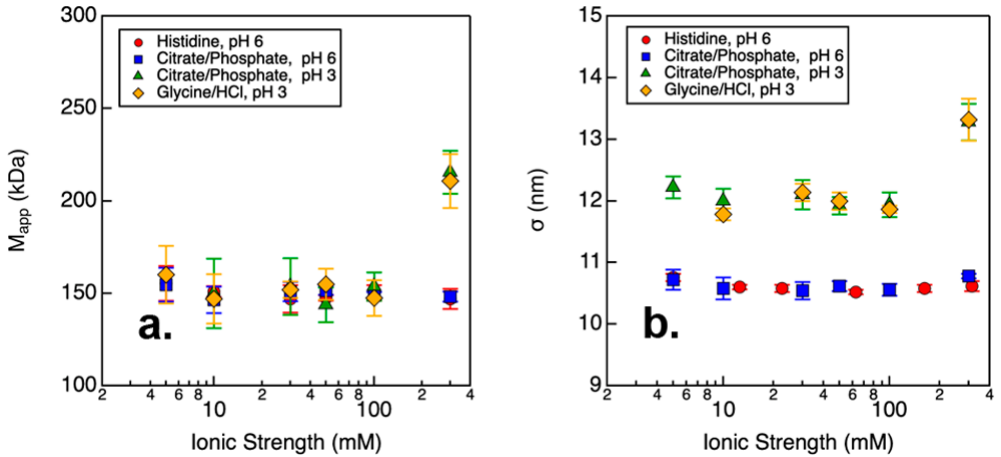
(a) *M*_app_ and (b) σ results measured from NISTmAb samples prepared in solutions with varying pH and ionic strength. Error bars correspond to one standard deviation from repeated measurements.

SLS and DLS were used to characterize the effects of pH and ionic strength on the size distribution of NISTmAb by measuring the average or apparent molecular weight *M*_app_ (Figure 1a) and the protein diameter σ (Figure 1b), respectively. In particular, pH 6 buffers were prepared with either histidine/histidine chloride or citrate/phosphate, while pH 3 buffers were prepared with either glycine/HCl or citrate/phosphate. For most conditions, SLS results show NISTmAb remained monomeric with *M*_app_ ≈ 150 kDa, regardless of the buffering species used. For both citrate/phosphate and glycine/HCl buffers at pH 3 and 300 mM ionic strength, the value of *M*_app_ was ca. 1.5 times larger than the theoretical protein size, which suggests the presence of either protein oligomers or small amounts of reversible or irreversible protein aggregates, as a result of the self-association of NISTmAb molecules. For simplicity, oligomer and aggregates are referred to as high-molecular-weight species throughout this manuscript. By contrast, analysis of the protein diameter indicates that NISTmAb acquired two different conformations depending on the solution pH. At pH 6, the protein had a size of 10.8 nm regardless of the buffering species and ionic strength. In acidic pH, the size of NISTmAb was measured to be around 12.0 nm for those conditions where the protein was monomeric (i.e., with ionic strength less than 300 mM). These results suggest the antibodies may adopt an extended conformation. At pH 3 and 300 mM ionic strength, the measured σ value increased to 13.3 nm. Considering the values of *M*_app_, this result for σ may be attributed to the formation of small oligomers, as no large species were identified by DLS.

**Figure 2:**
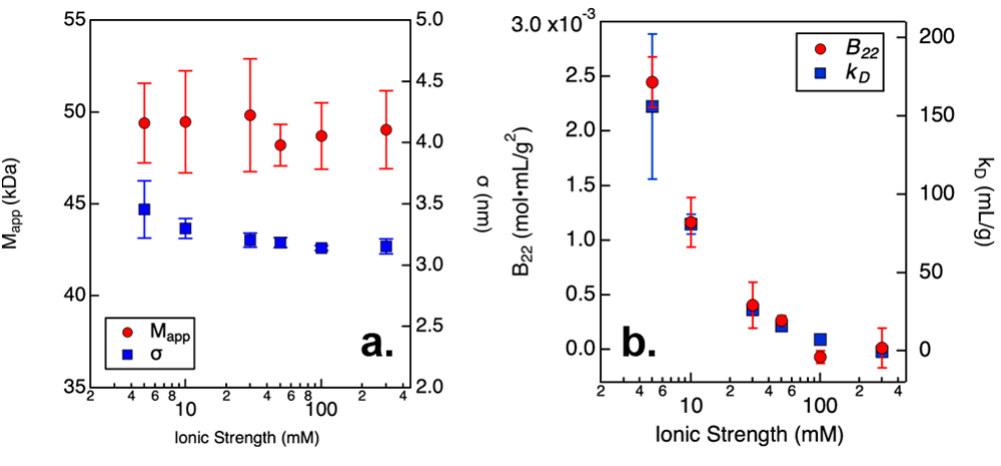
(a) *M*_app_ and σ values measured from Fab fragments prepared in citrate/phosphate buffer at pH 3 with varying ionic strength. (b) B_22_ and *k*_D_ values measured from Fab fragments prepared in citrate/phosphate buffer at pH 3 with varying ionic strength. Error bars correspond to one standard deviation from repeated measurements.

To identify the underlying cause for the anomalous behavior of NISTmAb under acidic conditions, SLS and DLS measurements were performed on the cleaved Fab fragment in citrate/phosphate buffer at pH 3 (Figure 2). The results show that the Fab was monomeric over the tested range of ionic strengths (5–300 mM), where the fitted *M*_app_ was statistically indistinguishable from the theoretical molecular weight of 47.6 kDa. Likewise, the resulting protein diameter σ was identical, within statistical error, for all of the evaluated conditions. In addition to *M*_app_ and σ, the *B*_22_ and *k*_D_ values were also obtained from the SLS and DLS measurements, respectively. Notably, none of these results suggest that the Fab domain was self-associating or forming irreversible aggregates, indicating that the associating behavior of the NISTmAb at low pH and high salt conditions was a consequence of interactions involving the Fc domain.

The physical stability of mAbs in solution is maintained by the balance between the repulsive and attractive forces between mAb molecules. One important type of interaction that affects the stability of mAbs is electrostatic interactions. Amino acids in proteins are zwitterions, meaning that they can be either positively or negatively charged depending on the buffer pH. In addition to pH, the presence of salt in solution can also modulate the interactions between mAb molecules by screening their surface charges. When protein surfaces carry a charge, they attract oppositely charged counterions from the surrounding solution through electrostatic interactions, creating a layer enriched with counterions called the Stern layer.^38^ The Stern layer is in close contact with the charged protein surface and moves together with the protein in solution. The Stern layer affects the effective charge *Z*_eff_ of the protein, which was measured by ELS and reflected its mobility in solution. Therefore, the presence of salt led to a reduced *Z*_eff_ of proteins due to the accumulation of counterions on their charged surfaces. Consequently, the electrostatic interactions between mAbs were reduced in the presence of salt, and this change might significantly impact the physical stability of mAbs in solution.

**Figure 3:**
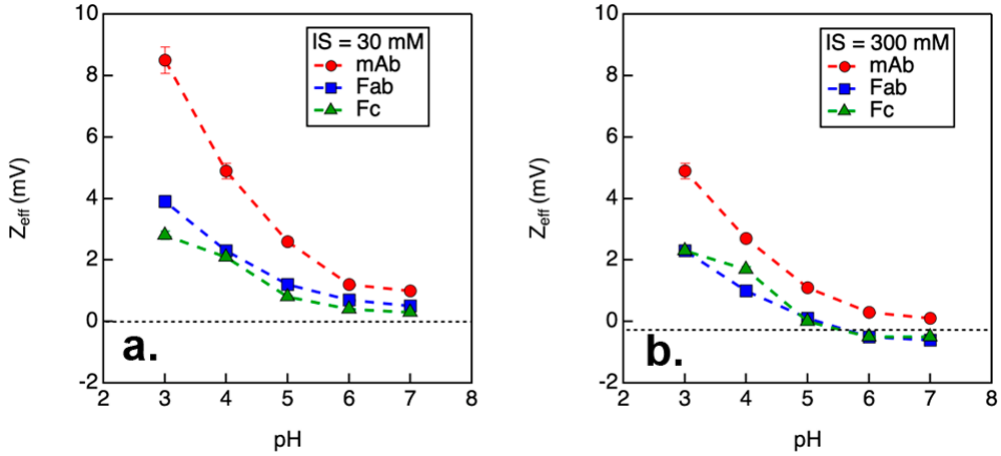
Effective charges measured from NISTmAb and its fragments with 30 and 300 mM ionic strength (IS). Samples were prepared in citrate/phosphate buffer. Error bars represent 5% of the measured results.

Theoretical calculations indicate that the isoelectric points (pI) of NISTmAb and its Fab and Fc fragments are at pH 8.5, 8.7, and 7.3,^19^ respectively. The *Z*_eff_ values measured from NISTmAb prepared at 30 mM ionic strength showed an increase with decreasing pH, with the lowest charge measured at pH 7 and the greatest charge measured at pH 3 (Figure 3). A similar trend was observed for the Fab and Fc fragments, although the *Z*_eff_ values measured from both fragments were systematically smaller than those measured from the full mAb. The reduction in *Z*_eff_ values at higher ionic strength can be explained by the screening effects of the additional salt ions, which reduce the electrostatic interactions between the protein molecules and counteract their charge. The *Z*_eff_ values measured at an ionic strength of 30 mM were in agreement with the theoretic pI values, as the mAb and fragments were expected to be neutral at pH above 8. However, with increasing ionic strength, the isoelectric points of the mAb and its fragments were reduced to pH 7 for the mAb and pH 5 for both Fab and Fc fragments, as shown in Figure 3. This change in pI can be explained by the accumulation of counterions at the Stern layer.^39,40^ Moreover, when compared to the *Z*_eff_ values measured at 30 mM ionic strength, the values measured at 300 mM ionic strength were significantly reduced at each pH condition.

It is also worth noting that the *Z*_eff_ values measured from Fab and Fc fragments were either neutral or slightly negative under conditions of 300 mM ionic strength and a pH value greater than 5. However, the apparent charge of NISTmAb demonstrated positive values despite the neutral or negative charge of the fragments. Such a discrepancy in charge between the full mAb and its individual fragments was observed only under high salt conditions and at a pH greater than 5. It is possible that the full mAb experienced different screening effects compared with the individual fragments. This could be due to constraints arising from the hinge region, leading to a specific geometry of the mAbs where the interaction of the ions with the protein surface was impeded.

### Secondary Structure of NISTmAb Characterized by CD and FTIR

**Figure 4:**
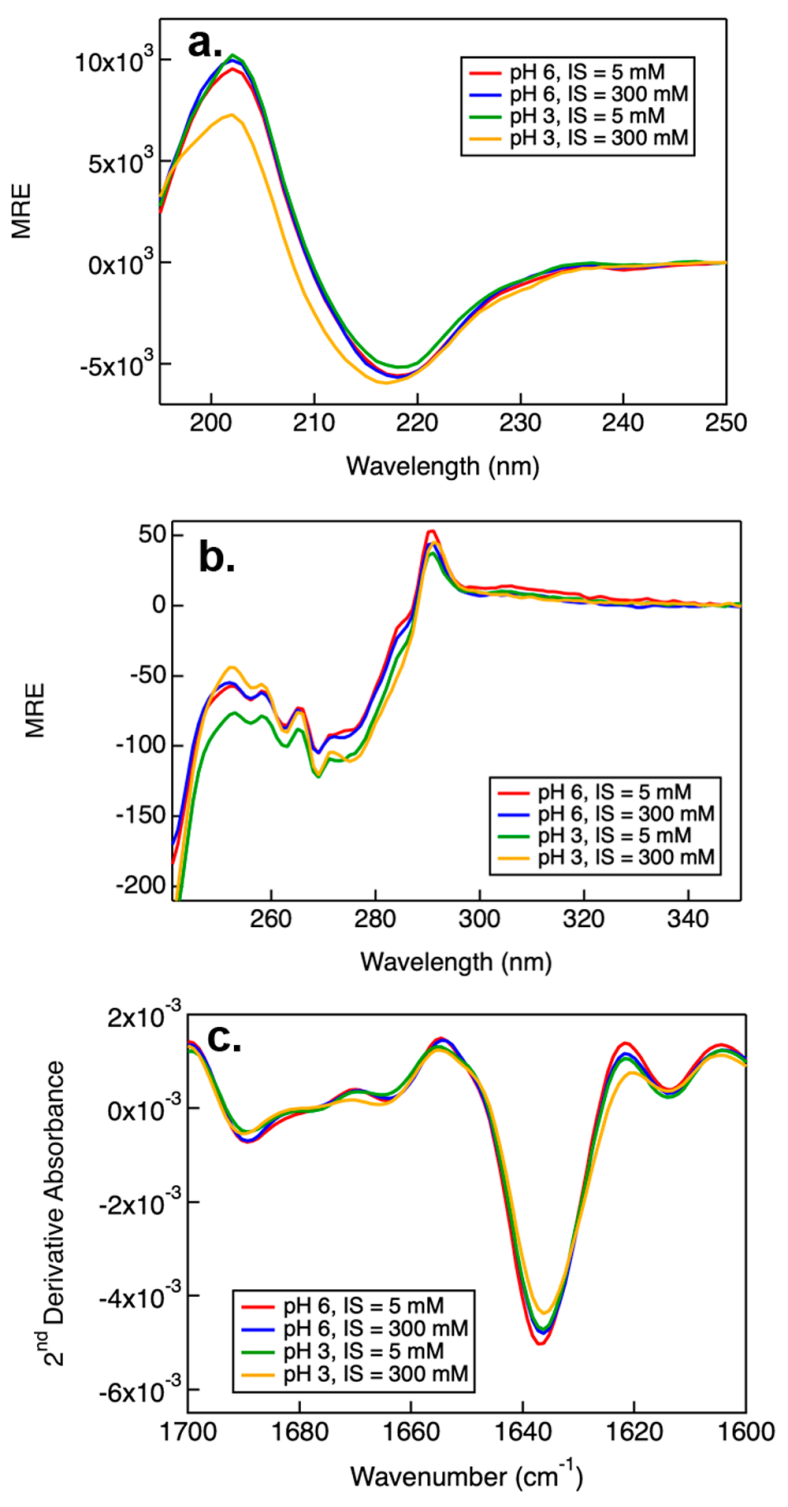
Far UV (a) and near UV (b) CD spectra and the second derivative of the FTIR spectra (c) measured from NISTmAb samples prepared in citrate/phosphate buffers, varying in pH and ionic strength (IS).

Circular dichroism (CD) and Fourier transform infrared (FTIR) spectroscopies were used to analyze the effects of pH and ionic strength on the protein conformation (Figure 4). To circumvent complexity arising from different buffering salts, samples were prepared in citrate/phosphate buffer but with varying pH and ionic strength. These techniques complement each other, as CD spectroscopy can measure α-helix, β-sheet, and random coil structures, while FTIR is more sensitive to β-sheet structures, which are commonly found in monoclonal antibodies.^41^ In the CD spectra, the mean residue ellipticity (MRE) was plotted against a wide range of wavelengths from 190 to 350 nm. The far-UV region of the CD spectra, depicted in Figure 4a, is sensitive to changes of protein secondary structure and provides insight into structural changes.^42,43^ The CD spectrum measured from NISTmAb sample prepared with a pH of 3 and an ionic strength of 300 mM appears different compared to other samples, indicating a minor decrease in β-sheet and an increase in random coil structures.^44^

The near-UV CD spectra of the NISTmAb samples are depicted in Figure 4b. Unlike the far-UV region, the near-UV part of the CD spectrum is capable of detecting changes in protein tertiary and quaternary structure.^42^ In this region, aromatic amino acids exhibit distinct wavelength profiles. Tryptophan has a peak near 290 nm and additional contributions between 290 and 305 nm, tyrosine has a peak between 275 and 282 nm, and phenylalanine has distinct bands between 255 and 270 nm.^45^ The changes in MRE seen in Figure 4b suggest differences in tertiary and quaternary structure between the samples, as previously reported.^46^ These changes in ellipticity are more pronounced in the near-UV between different pHs when compared to changes in ionic strengths, which indicate larger differences in the tertiary or quaternary structure. Previous DLS results (as shown in Figure 1b) suggested that the apparent diameter of NISTmAb increased when prepared at a low pH (when ionic strength was less than 300 mM). Thus, both DLS and CD results suggest an increase in the separation distance between the Fab and Fc fragments under low pH conditions, which may be due to the elevated net charges of the individual fragments (as shown in Figure 3). That is, at pH 3, the intramolecular domain–domain electrostatic repulsions resulted in an increased protein size and an extended conformation not observed in pH 6 buffer conditions.

Similar to the far-UV CD spectra, second derivative plots of the FTIR spectra of the six samples (Figure 4c) suggest that there were only small changes in the secondary structure among the different samples. Here, compared to other samples, the increase in coil structure observed by far-UV CD at low pH and high ionic strength is clearly seen as a decrease in the β-sheet conformation near 1638 cm^–1^. Mirroring the changes seen in the CD spectra, at low pH and high ionic strength, the IR shows that the amount of β-sheet content was reduced with a concomitant increase in random coil structure. Such secondary structure changes could be related to the formation of the high-molecular-weight species observed by both SLS and DLS earlier for samples prepared at pH 3 and 300 mM ionic strength.

### Effects of pH and Ionic Strength on the Conformation of NISTmAb Studied by SAXS

**Figure 5:**
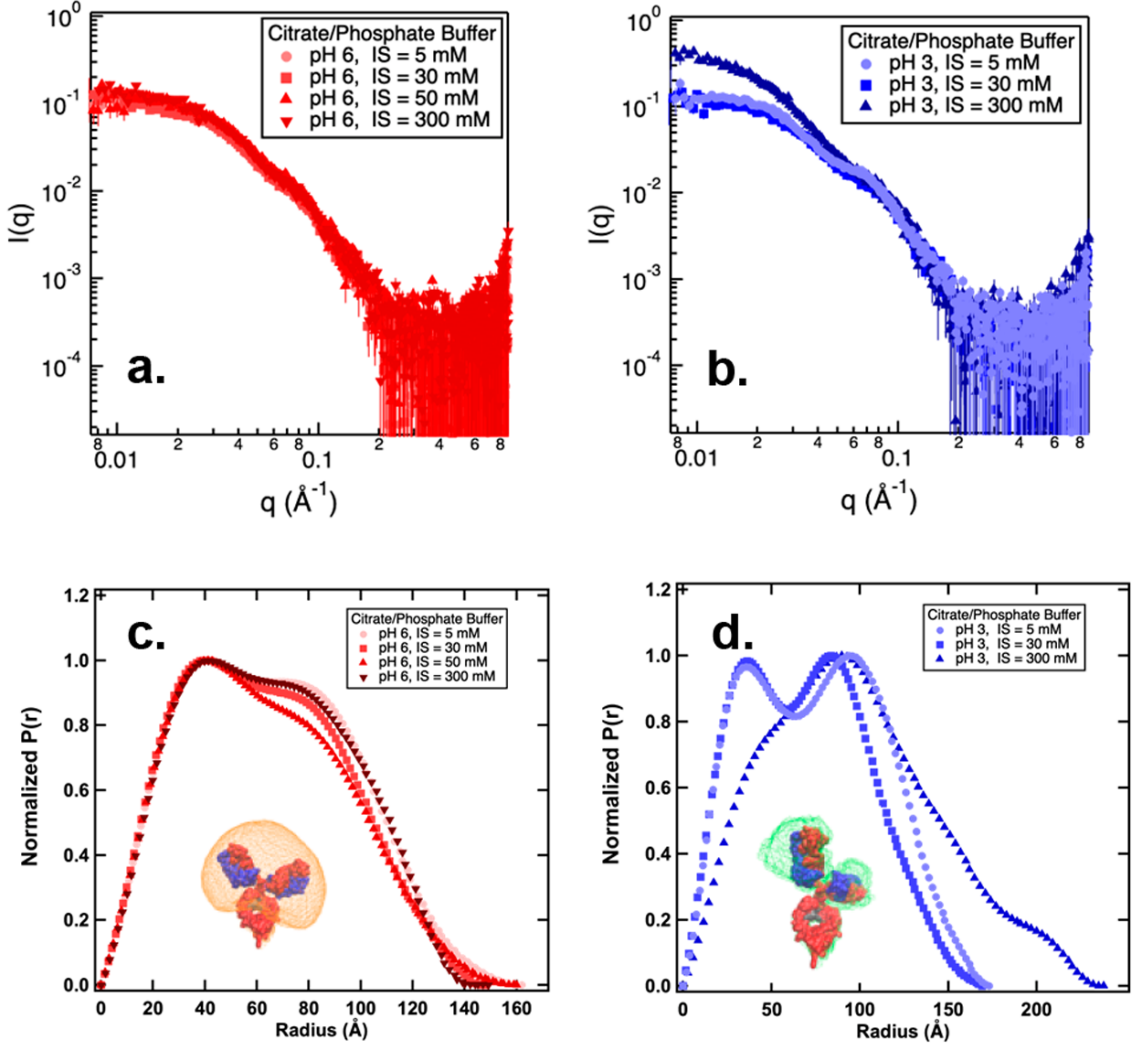
Scattering profiles measured from NISTmAb prepared in citrate/phosphate buffer at pH 6 (a) and at pH 3 (b). Samples were prepared with various ionic strength (IS) for both pHs. *P*(*r*) distribution functions derived from scattering profiles measured from citrate/phosphate buffer at pH 6 (c) and at pH 3 (d). Density plots representing the conformational space covered by Fab domains at pH 6 and pH 3 with ionic strength (IS) of 5 mM are also shown in (c) and (d) respectively. Error bars in scattering profiles (a and b) are propagated from the relative uncertainties in the scattering intensity measurements based on counting statistics. The statistical error bounding values correspond to 95% confidence limits.

The conformation of NISTmAb in different pH and ionic strength conditions was analyzed by using small-angle X-ray scattering (SAXS). Similar to the CD and FTIR measurements, the citrate/phosphate buffer system was used to prepare NISTmAb samples at both pH 6 and 3 to circumvent the possible effects coming from different buffering species. Figure 5a shows the scattering profiles obtained from 2 mg/mL NISTmAb samples prepared in a citrate/phosphate solution at pH 6. At this protein concentration, the interactions between mAb molecules were considered negligible,^34^ and thus the scattering profiles from these dilute samples were subjected to *P*(*r*) distribution analysis (Figure 5c and d). In general, all *P*(*r*) distribution functions exhibit two maxima. One at around 40 Å, which corresponds to the average size of the Fab and Fc fragments (i.e., the intradomain peak). The other maximum was observed at a distance of ∼80 Å, which indicates the spatial distance between the Fab and Fc (i.e., the interdomain peak).^30,47^ The peak position at 40 Å remained constant among all of the examined ionic strengths in the citrate/phosphate buffer at pH 6, implying that the conformation of the Fab and Fc fragments was preserved with increasing ionic strength. The position of the interdomain peak at 80 Å was also constant among all the examined ionic strengths, suggesting that the average spatial distance between the Fab and Fc did not change with ionic strength at pH 6. Previous research from our group evaluated the relative angles and distances between the Fab and Fc domains.^30^ It was found that the Fab-Fab and Fab-Fc distances were mostly within the 80–90 Å range, consistent with the observed interdomain peak position at 80 Å in the current study. Given the interdomain distance of 80 Å, it was anticipated that the domains were likely to be uniformly separated in pH 6 buffer conditions.^30^ At pH 6, the *D*_max_ value remained relatively constant despite varying ionic strengths, suggesting that the apparent size of the NISTmAb molecules was consistent under the examined buffer conditions. This result aligns with the DLS data, where a constant σ value was measured from samples prepared under pH 6 buffer conditions, as depicted in Figure 1b.

SAXS profiles of NISTmAb prepared in citrate/phosphate buffer at pH 3 are shown in Figure 5b, and the corresponding *P*(*r*) distributions are presented in Figure 5d. For samples prepared with an ionic strength less than 300 mM, the derived *P*(*r*) distribution profiles also feature two maxima. One at 35 Å, corresponding to the average size of the Fab and Fc fragments. This value is slightly smaller than that measured in citrate/phosphate buffer at pH 6. While the position of the intradomain peak remained constant at 35 Å, the interdomain peak decreased from 96 to 83 Å as the ionic strength increased from 5 mM to 30 mM, suggesting the spatial distances between the Fab and Fc domains were reduced with increased charge screening from the counterions. Moreover, the interdomain peak observed from the samples prepared at pH 3 was generally more pronounced than that measured at pH 6, implying that the NISTmAb molecules were less flexible at pH 3.

To better understand the flexibility of NISTmAb molecules in different pH environments, SAXS profiles measured at an ionic strength of 5 mM at both pH 3 and 6 were subjected to molecular simulation. SASSIE-web was used to generate Gaussian cube density profiles, which were visualized using VMD.^48^ The density plot is a representation of the volumetric space occupied by the atoms in an ensemble of structures, and it is derived by comparing theoretical scattering curves to the experimental SAXS data.^25,30^ It can be clearly seen from the density plot that the Fab occupied a larger volume of space when prepared at pH 6 with an ionic strength of 5 mM. At pH 3, the space occupied by the Fab decreased, suggesting that the mAb molecules were more rigid, with the Fab and Fc well separated from each other at pH 3. This result is in close agreement with the findings from the *P*(*r*) distribution analysis. At pH 3, with low to intermediate ionic strengths, NISTmAb as well as its fragments were highly charged (Figure 3), and thus the reduction in mAb flexibility could be from increased electrostatic repulsions between individual fragments. As evident from the *P*(*r*) distribution shown in Figure 5d, high-molecular-weight species were observed from NISTmAb at pH 3 with a 300 mM ionic strength. The intradomain peak was not present in the *P*(*r*) distribution profile, implying that the Fab and Fc fragments were no longer present as individual fragments, but instead had formed larger structures.

### Effects of pH and Ionic Strength on the PPI Studied by Light Scattering and SAXS

**Figure 6:**
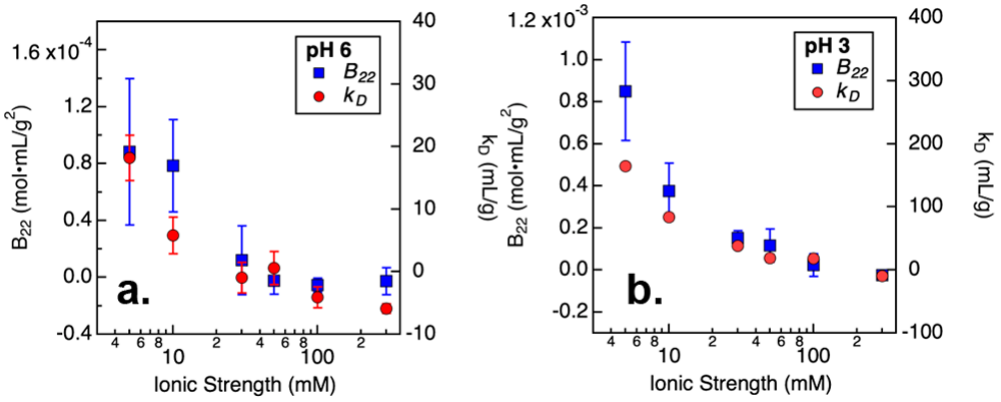
*B*_22_ and *k*_D_ values measured from NISTmAb prepared in citrate/phosphate buffer at pH 6 (a) and at pH 3 (b) with varying ionic strength. Error bars correspond to 95% confidence intervals for the fitted parameters.

In order to characterize PPI, Rayleigh scattering data as a function of protein concentration for NISTmAb at different solution conditions (i.e., pH and ionic strength) were obtained from SLS experiments as described in the Materials and Methods section. The different solution conditions allow us to probe the overall strength of the intermolecular interactions ranging from conditions where electrostatic forces are dominating (e.g., low pH and low ionic strength) to conditions where solvophobic interactions presumably control protein behavior (e.g., high pH and high ionic strength). In all examined buffer conditions, there was no indication of protein precipitation and visible particles. Measured Rayleigh scattering data was fitted to eq 1 to obtain the osmotic second virial coefficient (*B*_22_). Following a previous work,^22^ the range of protein concentration used for fitting Rayleigh scattering data was selected to ensure |*cB*_22_| ≤ 0.05 in order for eq 1 to be valid. Previous research show that a positive *B*_22_ value is indicative of net repulsive PPI, whereas a negative *B*_22_ value is indicative of net attractive PPI.^49^ Therefore, the *B*_22_ values shown in Figure 6 suggest that PPI for NISTmAb was mostly dominated by repulsive forces. With the lowest ionic strength, protein interactions were strongly repulsive, but their strength monotonically decreased with increasing ionic strength as a result of charge screening. Similarly, the strength of the interactions increased as pH moved away from the isoelectric point (pH = 9.18) from pH 6 to 3,^50^ due to increasing net protein charges. This type of qualitative behavior is generalized for most proteins, and it is anticipated that it was the charge–charge interactions dominated interprotein forces, yielding the behavior observed in Figure 6. Similar results on PPI were also obtained from the diffusion interaction parameter *k*_D_ measured by DLS. As described in the Materials and Methods section, both protein–protein and hydrodynamic interactions were captured from DLS through the change of the collective diffusion coefficient with respect to protein concentration at diluted conditions. In this context, positive values of *k*_D_ correspond to net repulsive interactions, while negative *k*_D_ values represent attractive interactions. Note that hydrodynamic forces generally lead to attractive interactions in protein solutions, and thus *k*_D_ might be negative when *B*_22_ indicates weakly repulsive interactions.^51,52^ The resulting values of *k*_D_ tell a similar story to that observed from *B*_22_. Upon examining the *B*_22_ and *k*_D_ values presented in Figure 6, it is evident that only when prepared at pH 3 with 300 mM ionic strength did both values (including the 95% confidence interval for both parameters) fall below the cutoff value between net attractive and net repulsive interactions (i.e., zero for *B*_22_ and −8 mL/g for *k*_D_(51−54)). Therefore, DLS and SLS results suggest that the overall PPI among NISTmAb molecules became net attractive under acidic and high salt conditions.

**Figure 7:**
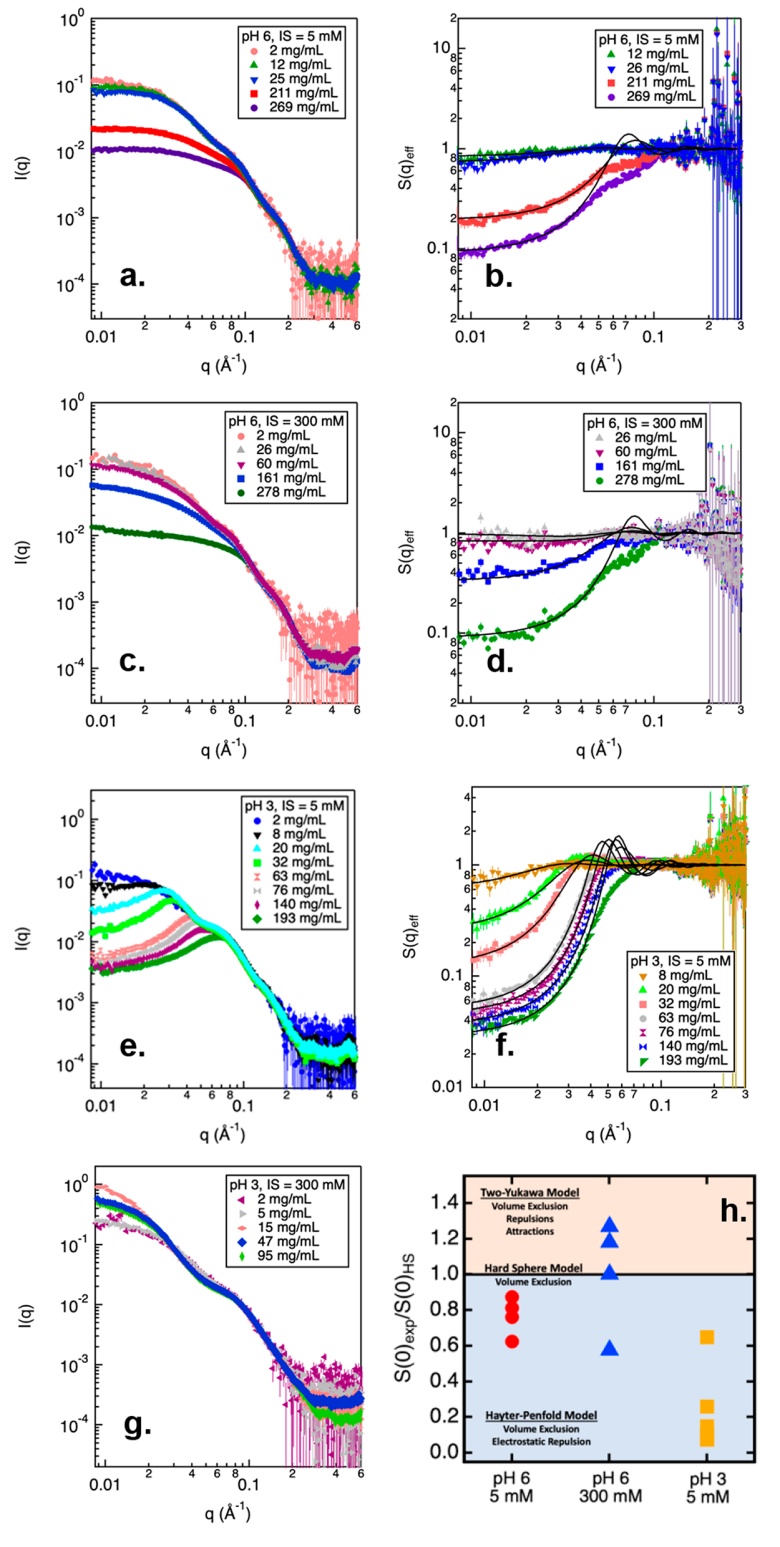
SAXS and *S*(*q*)_eff_ profiles measured from NISTmAb prepared in citrate/phosphate buffer at pH 6 and 3 with varying ionic strength (IS) and mAb concentrations (a–g). Error bars represent standard deviations calculated from the counting statistics. Figure (h) represents the *S*(0)_eff_/*S*(0)_HS_ values measured from NISTmAb samples prepared with varying pH, IS, and mAb concentration. Each data point in this figure represents the *S*(0)_eff_/*S*(0)_HS_ value obtained from a particular mAb concentration at the corresponding pH and ionic strength. In general, the *S*(0)_eff_/*S*(0)_HS_ value decreases with an increasing protein concentration. Shaded areas highlight different models used to fit the *S*(*q*)_eff_ profiles measured from different samples. Error bars in scattering profiles (a–g) are propagated from the relative uncertainties in the scattering intensity measurements based on counting statistics. The statistical error bounding values correspond to 95% confidence limits.

Small-angle X-ray/neutron scattering (SAXS/SANS) have been widely used in recent years to characterize PPI directly from concentrated mAb formulations.^26,34,55,56^ Therefore, in this study, the PPI of NISTmAb molecules was also characterized using SAXS (Figure 7). SAXS profiles of NISTmAb prepared in pH 6 buffer conditions are shown in Figure 7a and c. No upturn is observed in the low-*q* region from any of the SAXS profiles, indicating that there was no high-molecular-weight species formed in either 5 or 300 mM ionic strength at such a pH condition. Moreover, the scattering intensity measured from concentrated NISTmAb samples decreased toward the low-*q* region, suggesting that the net PPI among NISTmAb molecules were dominated by repulsive forces, similar to that observed from SLS/DLS results. Scattering profiles measured from samples prepared in pH 3 and 5 mM ionic strength also exhibit decreased scattering intensity toward the low-q region, suggesting that the overall PPI were net repulsive in acidic condition with low ionic strength. The *S*(*q*)_eff_ profiles measured from pH 6 buffer and pH 3 with 5 mM ionic strength all demonstrate a decreasing trend (become less than 1) as *q* decreases, suggesting that the overall PPI was of repulsive nature under these solution conditions. From the *S*(*q*)_eff_ profiles, we know that the overall PPIs were dominated by repulsions for samples prepared in pH 6 (both 5 and 300 mM) and pH 3 with 5 mM ionic strength. However, to better characterize the various intermolecular interactions contributed toward the net PPI among mAb molecules, the *S*(*q*)_eff_ profiles were fitted using appropriate models.

For NISTmAb samples prepared in buffers at pH 3 and 6 with low salt, the Hayter–Penfold model was used to fit the *S*(*q*)_eff_ profiles. The Hayter–Penfold model considers that the PPI are driven by volume exclusion and electrostatic repulsions. At low ionic strength, NISTmAb molecules were positively charged in pH 6 and 3 buffer (Figure 3), and therefore, it is not surprising that electrostatic repulsions were present between NISTmAb molecules under such conditions. At pH 6, but with a high ionic strength, the *S*(*q*)_eff_ profiles could not be fitted using a single model but varied with the mAb concentration. For NISTmAb concentrations less than 100 mg/mL, the Two–Yukawa model was used, suggesting the presence of both repulsive and attractive interactions between NISTmAb molecules. As the mAb concentration increased to 160 mg/mL, the hard sphere model provided the best fit for *S*(*q*)_eff_, suggesting a balance between attractive and repulsive forces, in addition to the repulsive forces arising from volume exclusion. Upon further increase in mAb concentration to 278 mg/mL, the Hayter–Penfold model was applied, suggesting PPI were driven by both volume exclusion and electrostatic repulsions. To better illustrate the nature of PPIs observed under different buffer conditions, we extrapolated *S*(0)_eff_ values from the fitted results. The *S*(0)_eff_ values obtained from NISTmAb samples prepared at pH 6 (with ionic strengths of 5 and 300 mM) and pH 3 (with an ionic strength of 5 mM) are presented in Table S2 in the Supporting Information. The *S*(0)_eff_ values measured from these samples are all less than 1, suggesting that the overall PPI among NISTmAb molecules is overall repulsive. To gain a better understanding of the nature of various interprotein interactions contributing to the overall repulsive PPI, the *S*(0)_eff_ values were compared to those derived from the hard sphere model (*S*(0)_HS_), which only considers volume exclusion effects.

Figure 7h summarizes the *S*(0)_eff_/*S*(0)_HS_ values obtained from all scattering profiles shown in Figure 7b, d, and f. If the *S*(0)_eff_/*S*(0)_HS_ ratio is less than 1, then the Hayter–Penfold model was employed to fit the *S*(*q*)_eff_ profile. Conversely, if the *S*(0)_eff_/*S*(0)_HS_ ratio is greater than 1, then the profile was fitted using the Two-Yukawa model. When the *S*(0)_eff_/*S*(0)_HS_ ratio equals 2, the hard sphere model was used to fit the *S*(*q*)_eff_ profile. At pH 6 with low ionic strength, the *S*(0)_eff_/*S*(0)_HS_ values measured from different mAb concentrations were all less than 1, suggesting that crowding effects did not alter the nature of PPI. However, at pH 6 and high ionic strength, NISTmAb molecules had a low effective charge (Figure 3), and the *S*(0)_eff_/*S*(0)_HS_ values showed dependence on mAb concentration. At low mAb concentration, the PPI contained both repulsive and attractive interactions, but as protein concentration increased, the *S*(0)_eff_/*S*(0)_HS_ values decreased to less than 1 suggesting that in highly crowded environments, the attractions between mAb molecules were diminished and dominated by repulsions.

At pH 3 and low ionic strength, the calculated *S*(0)_eff_/*S*(0)_HS_ values were the lowest among all examined conditions, which suggest that the PPI was strongly dominated by electrostatic repulsions. The repulsive PPI were likely due to the high charge of the NISTmAb molecules, resulting in the dominant contribution of electrostatic repulsions. As the ionic strength increased to 300 mM, NISTmAb molecules appeared to form high-molecular-weight species, as evident by the extended *D*_max_ from the *P*(*r*) distribution analysis. Charge measured at pH 3 and 300 mM ionic strength was lower than that measured from pH 3 and 30 mM, but still significantly greater than that measured from pH 6 conditions (Figure 3). However, even with such charge, NISTmAb molecules self-associated with form high-molecular-weight species even at dilute concentration, suggesting other types of attractive interactions also played a role in PPI observed in acidic and high salt conditions.

**Figure 8:**
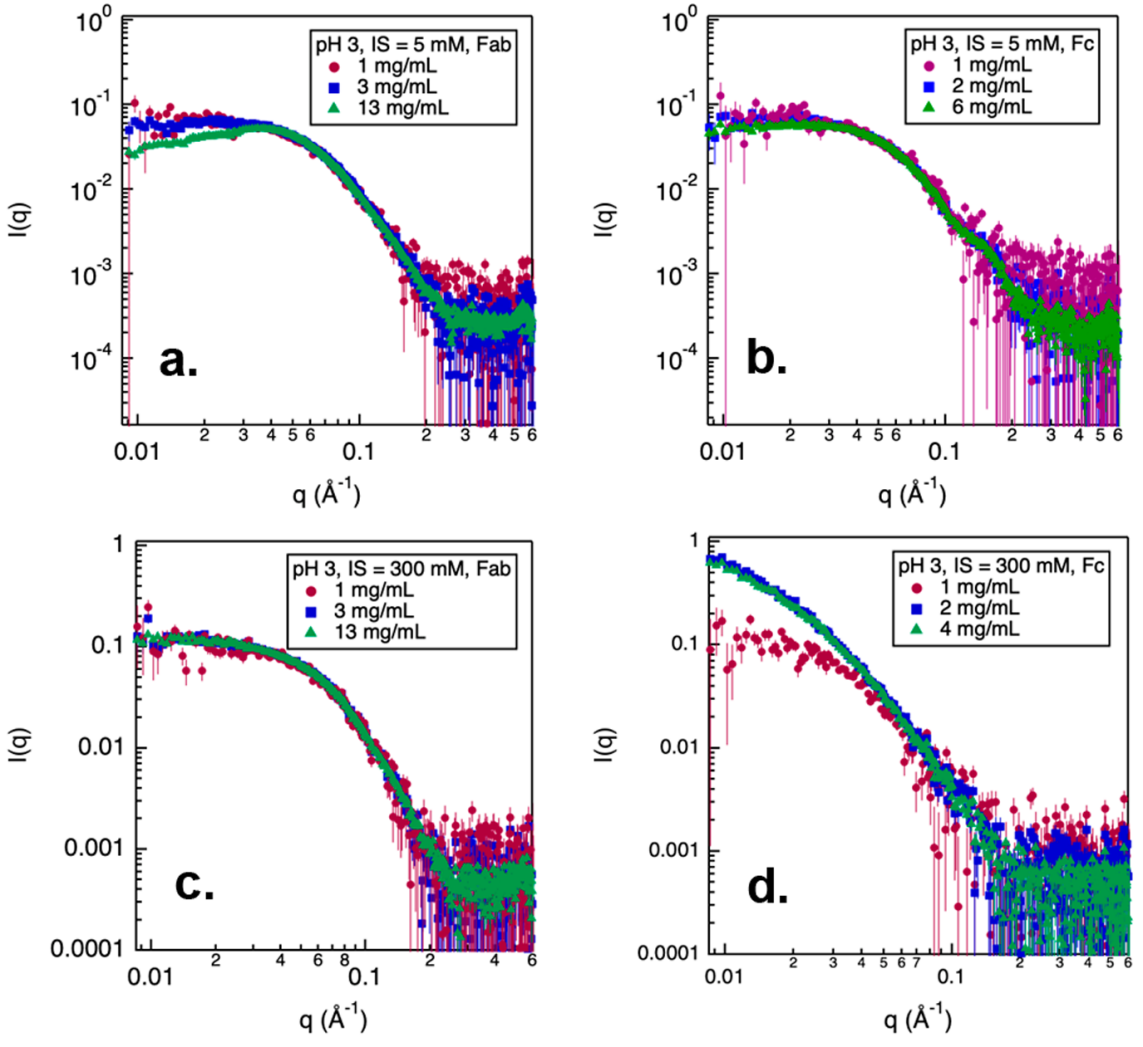
SAXS profiles measured from Fab and Fc in pH 3 buffer with varying ionic strengths (IS). Error bars in scattering profiles (a-d) are propagated from the relative uncertainties in the scattering intensity measurements based on counting statistics. The statistical error bounding values correspond to 95% confidence limits.

To investigate the origin of observed PPI among mAb molecules at pH 3, Fab and Fc samples were produced by digesting the full mAb and prepared in pH 3 buffers with ionic strengths of 5 mM and 300 mM. The SAXS profiles for the Fab and Fc at pH 3 and low ionic strength are shown in Figure 8a and b, respectively. At pH 3 and 5 mM, net repulsive PPI were observed in Fab samples at 13 mg/mL. For Fc, net repulsive PPI were observed at a concentration of 6 mg/mL. Therefore, at pH 3 and low ionic strength conditions, both Fab and Fc contributed toward the strong repulsive interactions observed in the full mAb samples. At a high ionic strength of 300 mM, NISTmAb molecules formed high-molecular-weight species, as indicated by the extended *D*_max_ observed in the *P*(*r*) distribution analysis (Figure 5d).

The scattering profiles measured from Fab and Fc fragments at pH 3 and high ionic strength are shown in Figure 8c and d, respectively. The scattering profiles from Fab and Fc fragments at pH 3 and high ionic strength revealed that no PPI was present among Fab fragments at concentrations below 13 mg/mL, while Fc showed significant self-association even at a concentration of 2 mg/mL. Therefore, the observed association of mAb molecules at dilute concentrations under pH 3 and high ionic strength can be largely attributed to attractions among the Fc fragments. Additional SAXS measurements will be performed to characterize the PPI in concentrated Fab and Fc samples for a better understanding of the associative behavior of NISTmAb observed at higher concentrations.

## Discussion

This study presented data on the conformation and PPI of NISTmAb when prepared under near neutral (pH 6) and acidic (pH 3) buffer conditions, with additional effects from ionic strength at each pH. Research on the effects of pH and ionic strength on mAb behavior is critical for enhancing our understanding of the factors that impact the stability of mAb products. Although the effects of pH and ionic strength on mAb aggregation behavior have been studied in the past, a general understanding is still limited, and several aspects of this complex phenomenon remain unclear.^57−61^ Previous studies have reported that the Fc regions of mAbs are more susceptible to unfolding at low pH, which can lead to increased aggregation.^57,58^ However, recent studies have also shown that the backbone torsion angles in the hinge region have larger degrees of freedom that allow for the Fab domains to adopt a larger set of structures that could help to improve protein stability by shielding access to the hydrophobic patches on the Fc.^30,62−64^ Therefore, a better understanding of the effects of pH and ionic strength requires insight into the flexibility of mAbs in various buffer conditions. In this study, we sought to examine the synergetic effects of pH and ionic strength on the conformational flexibility and PPI of NISTmAb molecules by using a range of biophysical characterization methods. Moreover, we prepared the Fab and Fc fragments and examined their physical properties to understand the contributions of each fragment toward the overall PPI in full mAb molecules.

Our results demonstrate that NISTmAb primarily exists as a monomer in most buffer conditions, except for pH 3 and 300 mM ionic strength, where high-molecular-weight species were observed. The diameter of NISTmAb measured by DLS remained constant at pH 6, while CD and FTIR analysis showed no significant structural changes of NISTmAb at this pH. Form factor analysis of the SAXS data indicates that the conformation of NISTmAb was not impacted by variations in ionic strength and that the NISTmAb molecules were flexible in all examined ionic strengths at pH 6. Similar results were reported from our previous study on NISTmAb, where a high degree of conformational flexibility was observed from the mAb molecules in histidine buffer at pH 6.^30^ Our results also show that at pH 6, as protein molecules become crowded in solution at high concentrations, the net PPI was dominated by repulsions, even though the net charge of the mAbs was relatively weak. It is possible that the high flexibility of the mAb molecules enabled the adoption of conformations that maximize electrostatic repulsions between mAb molecules.

Decreasing the pH of the buffer from 6 to 3 led to more noticeable effects of the ionic strength on NISTmAb. At low ionic strength, while the *M*_app_ measured from SLS remained similar to that measured at pH 6, the diameter of NISTmAb was larger. CD and FTIR analyses indicate that the secondary structure of NISTmAb was constant, whereas the tertiary structure changed slightly with low ionic strength. Further analysis of the SAXS data suggests that the flexibility of mAb molecules was significantly reduced under low pH and low ionic strength buffer conditions. The observed change in tertiary structure from CD and FTIR may result from NISTmAb molecules adopting a rigid and extended conformation, where the Fab fragments were at a greater distance from the Fc to accommodate strong electrostatic repulsions between individual domains. The PPI determined from NISTmAb, Fab and Fc fragments were all of a repulsive nature, suggesting that the interactions among NISTmAb molecules were dominated by electrostatic repulsions coming from both Fab and Fc domains.

At pH 3 with increased ionic strength, the surface charges of NISTmAb molecules were effectively screened by sodium and chloride ions, resulting in reduced electrostatic repulsions between the mAb molecules. With the decreased repulsion, NISTmAb molecules formed high-molecular-weight species, as evident by increased *M*_app_ and σ values from SLS and DLS measurements, respectively. The disappearance of the intradomain peak from the *P*(*r*) distribution profile indicates that the Fab and Fc domains of the mAb molecules no longer exist as individual domains, but rather they associate to form high-molecular-weight species. CD and FTIR analyses suggest that the secondary structure of NISTmAb was largely unchanged, although there is evidence to show that β-strand structures were unfolded into random coils. Domain-domain interactions measured from Fab and Fc reveal that the interactions among Fab were negligible, whereas self-association was observed from Fc even at dilute concentrations. Therefore, we hypothesize that at low pH and high ionic strength the self-association of NISTmAb was mainly driven by Fc-Fc attractions, although we cannot rule out Fab-Fc interactions leading to the observed behavior. Various interactions can contribute toward the PPI among mAb molecules, such as electrostatic interactions, hydrophobic interactions, hydrogen bonding, etc.^65^ Although both NISTmAb and its fragments were positively charged at pH 3, the charges on the protein surfaces were effectively screened by the high concentrations of NaCl, resulting in reduced electrostatic repulsions. Therefore, the roles played by hydrophobic interactions become more significant under such buffer conditions. Previous research has shown that the Fc fragment has more hydrophobic patches that are considered as “hot spots” with a high propensities for self-association. Thus, although both Fab and Fc had similar surface charges at pH 3 and 300 mM ionic strength, the Fc domains may have experienced more hydrophobic attractions between each other, leading to the Fc-Fc attractions that likely drove the observed self-association of NISTmAb in acidic pH with high salt.

## Conclusions

In this study, the conformational flexibility and protein–protein interactions (PPI) of NISTmAb were examined under near-neutral and acidic buffer conditions, each with varying ionic strength. Overall, NISTmAb was found to be physically stable under most conditions, except in acidic solutions with high salt, where high-molecular-weight species were observed. At pH 6, the protein exhibited a high flexibility, which may account for the observed net repulsive PPI at higher protein concentrations. In acidic conditions with low ionic strength, the flexibility of NISTmAb was significantly reduced, and the protein adopted an extended conformation. Both the Fab and Fc domains were highly charged under such buffer conditions, resulting in a strong repulsive PPI among the NISTmAb molecules. At pH 3, but with increased ionic strength, mAb molecules formed high-molecular-weight species, driven by the self-association of the Fc fragments due to increased hydrophobic interactions allowed as a result of charge screening. These findings highlight the significant roles of both conformational flexibility and domain–domain interactions in modulating the PPI of NISTmAb under acidic buffer conditions.

In addition to the detailed characterization of the effects of pH and ionic strength on the conformation and PPI of NISTmAb in solution, this study highlights the powerful combination of SAXS with DLS/SLS, CD and FTIR to obtain various molecular-level information (Table S1 in the Supporting Information). Our study shows that the results obtained from these experiments are in close agreement with each other, and each technique provides a unique piece of information that complements the others, leading to a more complete picture of the protein behavior in solution. Moreover, SAXS measurements offer additional information regarding the PPI among mAb molecules directly from concentrated formulations. Collectively, this study demonstrates the novel use of SAXS in combination with various biophysical techniques for the advanced characterization of therapeutic proteins, benefiting the rational design of stable biological formulations.

## Supporting Information Available

Summary of all experimental results measured from NISTmAb prepared at pH 3 and pH 6 under both low and high salt conditions; Fitting parameters for the different structure factor models used to fit the *S*(*q*)_eff_ profiles measured from different samples; Guinier fits of the scattering profiles measured from diluted samples prepared in various pH and ionic strength conditions (PDF)
