# Supplementary material for: Role of Domain–Domain Interactions on the Self-Association and Physical Stability of Monoclonal Antibodies: Effect of pH and Salt
Source: J Phys Chem B. 2023 Sep 26;127(39):8344–57. doi: 10.1021/acs.jpcb.3c03928 (PMC10561141; doi:10.1021/acs.jpcb.3c03928)
Supplement: Supplementary file 1 — jp3c03928_si_001.pdf [file jp3c03928_si_001.pdf]

Role of Domain-Domain Interactions on the Self-association and Physical Stability of Monoclonal Antibodies: Effect of pH and Salt

Amy Y. Xu[1*], Marco A. Blanco[2], Maria Monica Castellanos[3,4], Curtis W. Meuse[3,5], Kevin Mattison[6], Ioannis Karageorgos[3, 5], Harold W. Hatch[7], Vincent K. Shen[7] and Joseph E. Curtis[4*]

[1] Department of Chemistry, Louisiana State University, Baton Rouge, LA 70803, USA

[2] Discovery Pharmaceutical Sciences, Merck Research Laboratories, Merck & Co., Inc, West Point, PA 19486, USA

[3] Institute for Bioscience and Biotechnology Research, University of Maryland, Rockville, MD 20850, USA

[4] NIST Center for Neutron Research, National Institute of Standards and Technology, Gaithersburg, MD 20899, USA

[5] Biomolecular Measurement Division, National Institute of Standards and Technology, Gaithersburg, MD 20899, USA

[6] Malvern Panalytical, Westborough, MA 01581, USA

[7] Chemical Sciences Division, Material Measurement Laboratory, National Institute of Standards and Technology, Gaithersburg, MD 20899, USA

Corresponding Authors:
*Amy Y. Xu: amyxu@lsu.edu
*Joseph E. Curtis: joseph.curtis@nist.gov

Table S1. Summary of all experimental results measured from NISTmAb prepared at pH 3 and pH 6 under both low and high salt conditions. Results leading to similar conclusions are highlighted using the same shade of color.

	**Protein Conformation**			
	**pH 6, 5 mM**	**pH 6, 300 mM**	**pH 3, 5 mM**	**pH 3, 300 mM**
**Far-UV CD**	Similar spectra were obtained under these conditions, suggesting that the secondary structure of NISTmAb remained consistent. (In agreement with FTIR results)			There was a decrease in β-sheet content and an increase in random coil content. (In agreement with FTIR results)
**Near-UV CD**	Similar spectra were obtained, indicating that the tertiary/quaternary structure of NISTmAb remains consistent regardless of the ionic strength at pH 6. (In agreement with SAXS results)		The difference in MRE could result from the extended conformation. (In agreement with DLS and SAXS results)	The difference in MRE could be resulted from the formation of high-molecular-weight species (In agreement with DLS, SLS and SAXS results)
**FTIR**	Similar spectra were obtained under these conditions, suggesting that the secondary structure of NISTmAb remained consistent. (In agreement with far-UV results)			There was a decrease in β-sheet content and an increase in random coil content. (In agreement with far-UV results)
**DLS**	σ value suggests NISTmAb was monomeric (In agreement with SLS, SAXS results)		An increased σ value suggests that NISTmAb was monomeric and exhibited an extended conformation. (In agreement with near-UV and SAXS results)	A significantly increased σ value suggests that NISTmAb might form high-molecular-weight species. (In agreement with near-UV CD, SLS and SAXS results)
**SLS**	M_{app} value suggests NISTmAb was monomeric (In agreement with SLS, SAXS results)			Increased M_{app} value suggests NISTmAb formed high-molecular-weight species (In agreement with near-UV CD, DLS and SAXS results)

	pH 6, 5 mM	pH 6, 300 mM	pH 3, 5 mM	pH 3, 300 mM
**SAXS**	Similar P(r) distribution profiles were obtained, suggesting that the overall conformation and apparent size of NISTmAb remained consistent at pH 6, regardless of ionic strength. (In agreement with near-UV CD, and DLS results)		The inter-domain peak shifted to a larger length-scale and became more pronounced, implying that the NISTmAb molecules were less flexible and adopted a conformation in which the Fab and Fc domains were more separated. (In agreement with near-UV CD and DLS results)	The significantly greater D_{max} and the disappearance of the intra-domain peak from the P(r) distribution profile suggest that NISTmAb formed high-molecular-weight species (In agreement with near-UV CD, SLS and DLS results)
**Overall Protein-Protein Interactions (PPI)**				
	**pH 6, 5 mM**	**pH 6, 300 mM**	**pH 3, 5 mM**	**pH 3, 300 mM**
**DLS**	Net repulsive (In agreement with SLS and SAXS results)			Net attractive (In agreement with SLS and SAXS results)
**SLS**	Net repulsive (In agreement with DLS and SAXS results)			Net attractive (In agreement with DLS and SAXS results)
**SAXS**	Net repulsive (In agreement with DLS and SLS results)			Net attractive (In agreement with DLS and SLS results)
**Nature of various inter-protein interactions contribute to the repulsive PPI**				
	**pH 6, 5 mM**	**pH 6, 300 mM**	**pH 3, 5 mM**	**pH 3, 300 mM**
**SAXS**	Volume Exclusion Electrostatic Repulsion (In line with zeta potential results)	Varies with mAb concentration (In line with zeta potential results)	Volume Exclusion Electrostatic Repulsion (In line with zeta potential results)	N/A

Table S2. Fitting parameters for the different structure factor models used to fit the $S(q)_{eff}$ profiles measured from different samples.

**HAYTER-PENFOLD MODEL**				
**pH 6, IS = 5 mM**				
mAb Concentration	12 mg/mL	26 mg/mL	211 mg/mL	269 mg/mL
Diameter (Å)	80	85	71	70
Charge	5.6	5.7	3.6	6.4
$S(0)_{eff}$	0.80	0.68	0.18	0.093
**pH 6, IS = 300 mM**				
mAb Concentration	278 mg/mL			
Diameter (Å)	80			
Charge	60			
$S(0)_{eff}$	0.086			
**pH 3, IS = 5 mM**				
mAb Concentration	8 mg/mL	20 mg/mL	32 mg/mL	63 mg/mL
Diameter (Å)	88	77	73	73
Charge	21	29	36	83
$S(0)_{eff}$	0.61	0.23	0.12	0.050
mAb Concentration	76 mg/mL	140 mg/mL	193 mg/mL	
Diameter (Å)	72	81	85	
Charge	94	51	45	
$S(0)_{eff}$	0.044	0.035	0.029	
**HARD SPHERE MODEL**				
**pH 6, IS = 300 mM**				
mAb Concentration	161 mg/mL			

Radius (Å)	39	
$S(0)_{eff}$	0.33	
**TWO-YUKAWA MODEL pH 6, IS = 300 mM**		
mAb Concentration	26 mg/mL	60 mg/mL
Radius (Å)	48	43
K_1	6.0	6.0
Z_1	10	10
K_2	-3.0	-3.3
Z_2	9.6	8.2
$S(0)_{eff}$	0.98	0.84

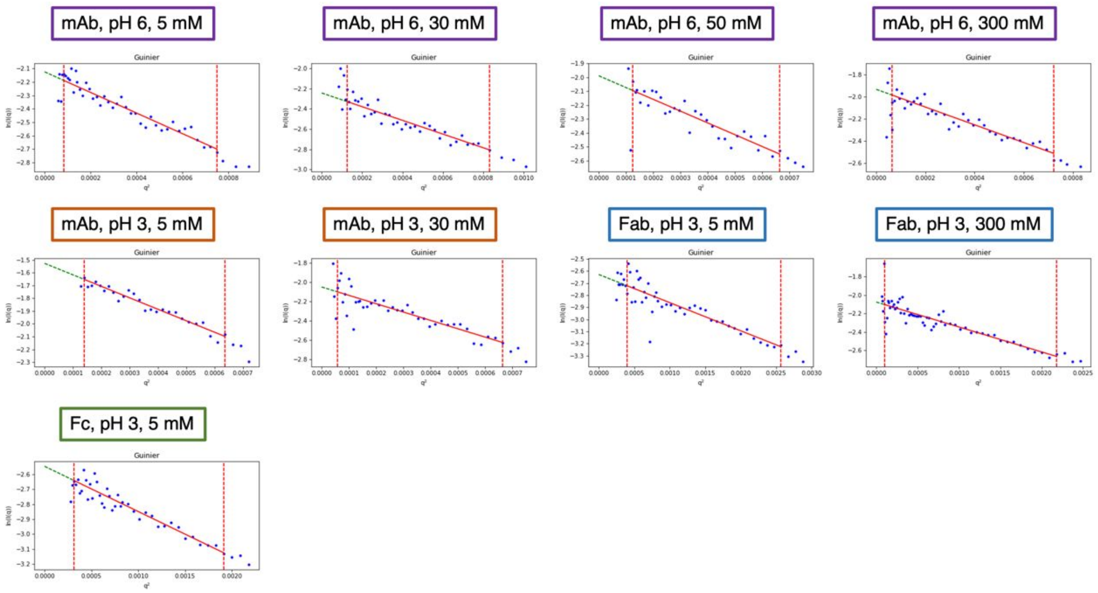

Figure S1. Guinier fits of the scattering profiles measured from diluted samples prepared in various pH and ionic strength conditions. The software BioXTAS RAW was used to generate these plots[1]

References:

1. Hopkins, J. B.; Gillilan, R. E.; Skou, S., BioXTAS RAW: improvements to a free open-source program for small-angle X-ray scattering data reduction and analysis. *Journal of Applied Crystallography* **2017,** *50*, 1545-1553.
